# The complete chloroplast genome of leguminous forage *Onobrychis viciifolia*

**DOI:** 10.1080/23802359.2021.1886017

**Published:** 2021-03-15

**Authors:** Xiao Fu, Xiuyun Ji, Bianbian Wang, Lei Duan

**Affiliations:** a Ningxia Yunwu Mountains National Natural Reserve, Guyuan, China; b Key Laboratory of Plant Resources Conservation and Sustainable Utilization, South China Botanical Garden, Chinese Academy of Sciences, Guangzhou China

**Keywords:** Chloroplast genome, *Onobrychis viciifolia*

## Abstract

*Onobrychis viciifolia* is mainly distributed in Europe and has been widely cultivated in North and Northwest of China. The complete chloroplast genome was sequenced using the Illumina Hiseq X-Ten platform. The genome lacks an inverted repeat (IR) region, containing 76 protein-coding genes, 30 tRNAs genes and 4 rRNAs. The overall GC content is 34.6%. A phylogenetic tree based on the whole chloroplast genomes of 14 species indicated that *Onobrychis viciifolia* belonged to the tribe Hydysareae in IRLC group of the subfamily Papilionoideae (Leguminosae), and it was sister to the genus *Hedysarum*.

*Onobrychis viciifolia* is a deep-rooted perennial legume with erect or sub-erect hollow stems growing up to a height of 100 cm (Frame et al. 1998). As a high quality forage crop, it has been cultivated in Northwest China for a long period (Xu 1998). Previous studies reported that *Onobrychis viciifolia* has good tolerance to drought-stress, salt-stress (Beyaz et al. 2017; Beyaz 2019), a high nutritive value, high voluntary intake and palatability to grazing animals (Bhattarai et al. 2016), but wild resources has been barely witnessed. A good knowledge in genomic information of this species would contribute to the study on its molecular studies, geographical distribution, diversity, breeding and fodder production.

The fresh leaves of *Onobrychis viciifolia* was collected in Yunwu Mountains National Natural Reserve, Ningxia, China (106°23′15″E, 36°15′38″N), and the voucher specimen was deposited in the herbarium of South China Botanical Garden, Chinese Academy of Sciences (IBSC, collection #: L.Duan 2018014). We extracted the total genomic DNA with CTAB approach (Doyle 1987), the genomic libraries were prepared and sequenced using the Illumina Hiseq X-Ten platform (Illumina Inc. San Diego, CA). The resultant sequences were filtered following Yao et al. (2016), the adaptor-free reads were then assembled with SPAdes 3.11 (Bankevich et al. 2012). We annotated the assembly of complete chloroplast (cp) genomes using the Dual Organellar GenoMe Annotator (DOGMA) (Wyman et al. 2004) and deposited the genomes in GenBank (accession number: MW007721).

About 1.09 Gb raw reads of *Onobrychis viciifolia* were obtained, with coverage of 918× and 121,932 bp in length. The cp genome lacked inverted repeat (IR) region. The genome contained 76 protein-coding genes (CDS), 30 transfer RNA genes(tRNA), 4 ribosomal RNA genes (rRNA), within which 16 genes (*ndhA, ndhB, rpl2, rpl16, petD, petB, clpP, rps12, atpF, rpoC1, trnG-UCC, trnA-UGC, trnI-GAU, trnL-UAA, trnV-UAC and trnK-UUU)* had one intron, 2 gene (*rps*12, *ycf*3) has two introns, respectively. Overall GC content of the whole genome was 34.6%.

To infer the phylogenetic relationships among this species and its related taxa, whole cp genomes of 13 Papilionoideae species were downloaded from GenBank, which were aligned with that of *Onobrychis viciifolia* by applying MAFFT v.7 (Katoh and Standley 2013). Based on the alignment, a aximum-likelihood (ML) tree was constructed using IQ-TREE v.1.6 (Nguyen et al. 2015). The result (Figure 1) showed that *Onobrychis viciifolia, Hedysarum petrovii, Hedysarum taipeicum* and *Alhagi sparsifoli*a constituted a well-supported tribe Hydysareae [as in Duan et al. (2020)], serving as the sister of the *Hedysarum* genus. This tribe was a member of the inverted repeat-lacking clade (IRLC), which in turn belonged to the clade of Hologalegina in papilionoid legumes as suggested by previous studies (Wojciechowski et al. 2004; Schrire 2005).

**Figure 1.**
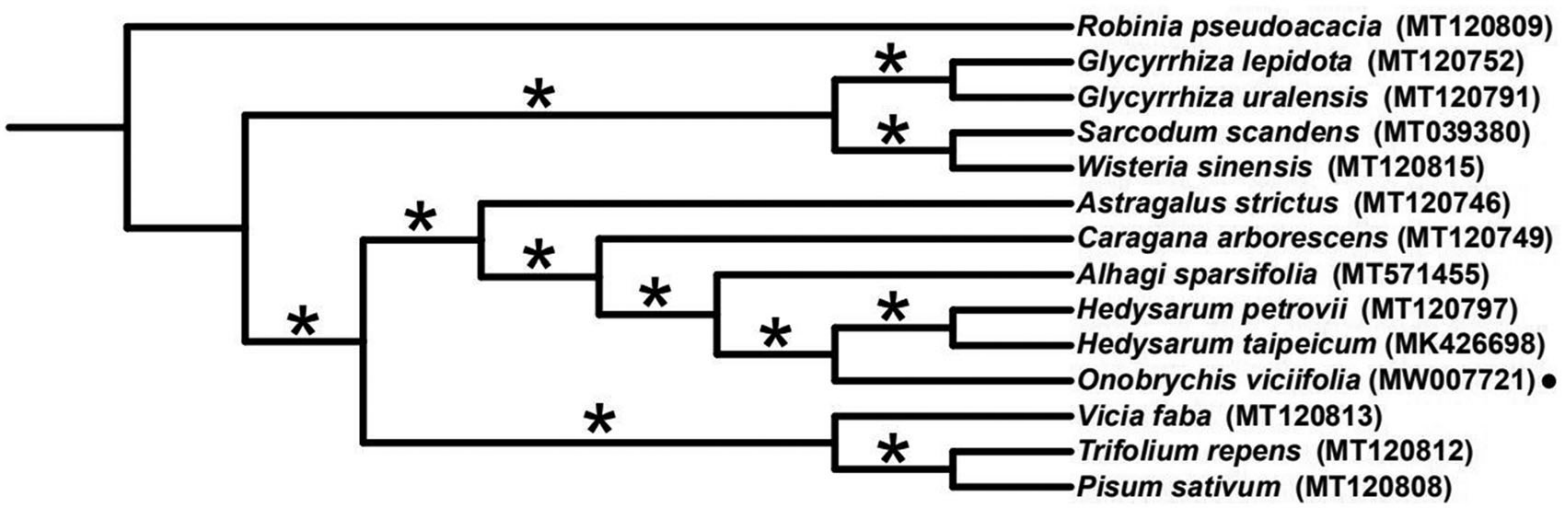
Maximum likelihood (ML) phylogenetic tree based on 14 chloroplast genomes of Fabaceae. The position of *Onobrychis viciifolia* is indicated with black dot. The bootstrap values of 100% are shown on branches with asterisks.

## Data Availability

The voucher specimen was deposited in the herbarium of South China Botanical Garden, Chinese Academy of Sciences (IBSC, collection #: L.Duan 2018014) (http://herbarium.scbg.cas.cn/). To infer the phylogenetic relationships among this species and its related taxa, whole cp genomes of 13 Papilionoideae species were downloaded from GenBank (https://www.ncbi.nlm.nih.gov/). The genome sequence data that support the findings of this study are openly available in GenBank of NCBI at (https://www.ncbi.nlm.nih.gov/) under the accession no. MW007721. The associated BioProject, SRA, and Bio-Sample numbers are PRJNA690644, SRR13385163, and SAMN17255786, respectively (https://www.ncbi.nlm.nih.gov/sra/?term=SRR13385163).
